# Case Report: Neuronal intranuclear inclusion disease mimicking recurrent stroke in the setting of intracranial stenosis

**DOI:** 10.3389/fmed.2026.1838444

**Published:** 2026-05-20

**Authors:** Jingmin Zhao, Guangxun Shen, Lumei Chi

**Affiliations:** Department of Neurology, China-Japan Union Hospital of Jilin University, Changchun, China

**Keywords:** diffusion magnetic resonance imaging, intracranial atherosclerosis, neuronal intranuclear inclusion disease, skin biopsy, stroke mimics

## Abstract

**Background:**

Neuronal intranuclear inclusion disease (NIID) is a rare, progressive neurodegenerative disorder that can present with stroke-like episodes, posing a significant diagnostic challenge. This difficulty is compounded when it coincides with intracranial atherosclerotic stenosis, a common cause of recurrent stroke.

**Case presentation:**

We report the case of a 64-years-old woman without conventional vascular risk factors who presented with a recurrent acute focal neurological deficit–her sixth similar episode over 8 years, each previously diagnosed as cerebral infarction. Brain magnetic resonance imaging (MRI) revealed a new area of restricted diffusion in the right frontoparietal region and an ipsilateral middle cerebral artery (MCA) M2 segment stenosis (>50%). Crucially, a systematic review of the diffusion-weighted imaging (DWI) sequences identified the pathognomonic linear hyperintensity along the corticomedullary junction, known as the “ribbon sign,” which is highly suggestive of NIID. Despite a negative genetic test for the NOTCH2NLC GGC repeat expansion, the diagnosis was confirmed by a minimally invasive skin biopsy, which demonstrated characteristic non-membranous intranuclear inclusions on electron microscopy.

**Conclusion:**

We highlight the imperative to recognize this specific and pathognomonic neuroimaging pattern even in the presence of coincidental atherosclerosis. Furthermore, we detail the systematic diagnostic approach when genetic testing is unrevealing, emphasizing the pivotal role of targeted tissue biopsy in achieving a definitive diagnosis. This report underscores the key learning points in evaluating acute stroke mimics with atypical evolution.

## 1 Introduction

The differential diagnosis of acute focal neurological deficits encompasses not only ischemic stroke but also a range of “stroke mimics,” among which neurodegenerative disorders pose particular diagnostic difficulty. Neuronal intranuclear inclusion disease (NIID) is a progressive multisystem proteinopathy defined by eosinophilic intranuclear inclusions in both central and peripheral tissues (1). Its heterogeneous clinical spectrum includes episodic encephalopathy, cognitive decline, autonomic dysfunction, and stroke-like episodes. The latter presentation–often associated with transient diffusion restriction on MRI–may lead to repeated misdiagnosis as cerebral infarction, especially when coincident vascular abnormalities are present. We describe an instructive case of NIID masquerading as recurrent ischemic stroke and highlight key diagnostic clues and a practical diagnostic pathway for vascular neurologists.

## 2 Case report

A 64-years-old right-handed woman presented to our stroke unit with a 10-days history of progressive left-sided limb weakness and dysarthria. She reported five prior similar episodes over the past 8 years, each previously diagnosed as “cerebral infarction,” yet she had maintained excellent functional recovery (pre-morbid modified Rankin Scale score 1). She had no history of hypertension, diabetes, or hyperlipidemia. There was no family history of neurological disorders, including dementia, movement disorders, or recurrent stroke-like episodes. Neurological examination revealed dysarthria, left central facial palsy, left hemiparesis (Medical Research Council grade 3/5), and a left extensor plantar response (National Institutes of Health Stroke Scale score 6). The initial working diagnosis was recurrent acute ischemic stroke in the right middle cerebral artery territory.

### 2.1 Neuroimaging findings

**FIGURE 1:**
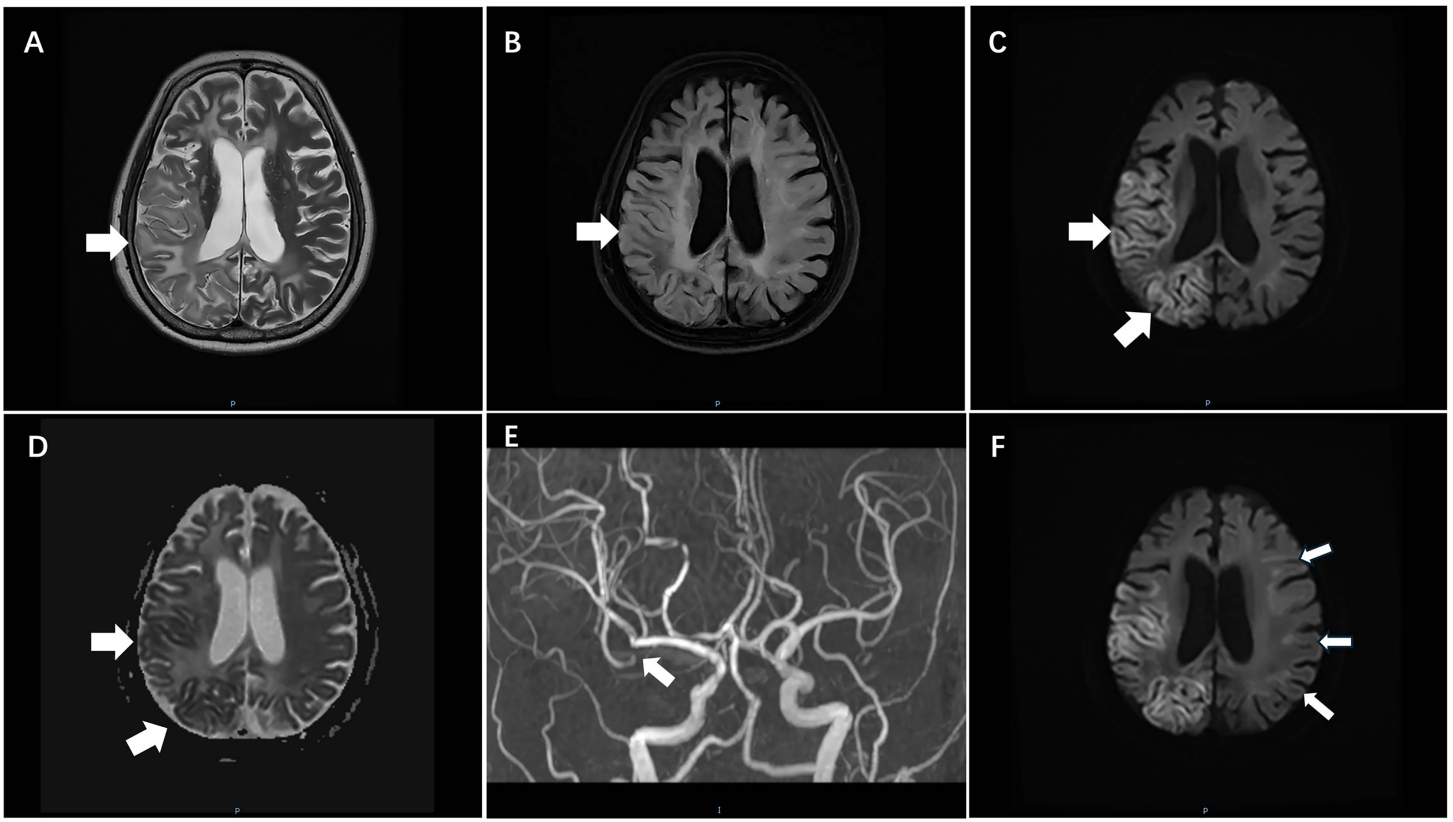
Neuroimaging features of a stroke-like episode in a patient with neuronal intranuclear inclusion disease (NIID). **(A)** Axial T2-weighted image shows a new, ill-defined area of cortical/subcortical hyperintensity in the right frontal-parietal region (arrow); **(B)** Fluid-attenuated inversion recovery (FLAIR) sequence confirms the hyperintense lesion in the same region (arrow); **(C)** Diffusion-weighted imaging (DWI) demonstrates corresponding bright signal (arrow), indicating restricted diffusion; **(D)** Apparent diffusion coefficient (ADC) map reveals hypointensity (arrow) in the same area; **(E)** Magnetic resonance angiography (MRA) demonstrates focal stenosis of the right middle cerebral artery M2 segment (arrow); **(F)** A DWI sequence at a different brain level reveals the pathognomonic sign of NIID: a continuous, linear hyperintensity along the corticomedullary junction (arrow).

Brain MRI demonstrated a cortical/subcortical hyperintensity in the right frontoparietal region on T2-weighted and fluid-attenuated inversion recovery sequences, with corresponding restricted diffusion (hyperintense on diffusion-weighted imaging and hypointense on the apparent diffusion coefficient map), consistent with subacute ischemia (Figures 1A–D). Magnetic resonance angiography revealed focal stenosis (>50%) of the right MCA M2 segment (Figure 1E). A crucial additional finding was a continuous linear hyperintensity along the corticomedullary junction on DWI–the pathognomonic “ribbon sign” of NIID (Figure 1F).

### 2.2 Differential diagnosis

The differential diagnosis for this patient included recurrent cerebral infarction due to M2 stenosis, MELAS syndrome, Creutzfeldt-Jakob disease (CJD), autoimmune encephalitis, and NIID. MELAS syndrome was excluded by genetic testing for mitochondrial DNA common mutations, which were negative. CJD was ruled out by electroencephalography, which showed no periodic sharp wave complexes (triphasic waves). Autoimmune encephalitis was excluded by negative serum autoantibody panels (including anti-NMDAR, anti-LGI1, anti-CASPR2, anti-GABABR, and anti-AMPAR). The recurrent, stereotyped episodes with near-complete recovery over 8 years, absence of conventional vascular risk factors, and the pathognomonic “ribbon sign” on DWI ultimately pointed to NIID as the most likely diagnosis.

### 2.3 Diagnostic work-up and management

**FIGURE 2:**
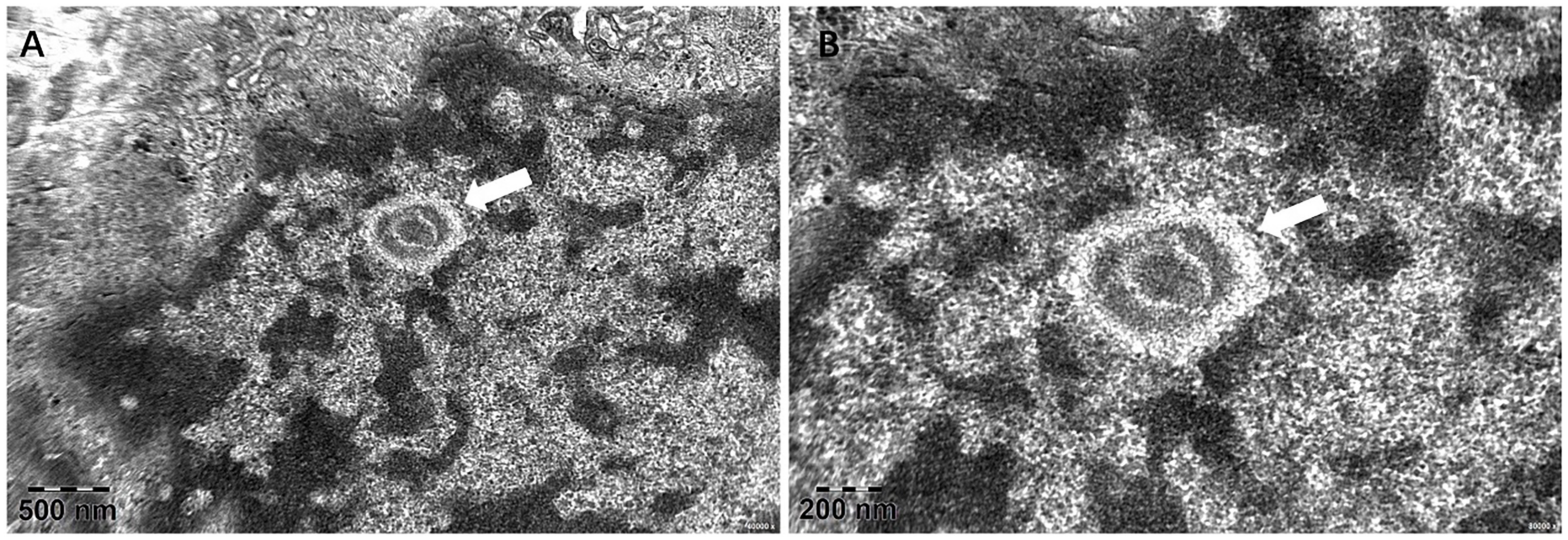
Electron microscopic examination of the skin biopsy. **(A)** Ultrathin section under electron microscopy shows intranuclear inclusions within a fibroblast. The inclusions are round, well-demarcated, and non-membranous. They exhibit low electron density and have a finely granular and fibrillar internal texture; **(B)** Image at a different magnification provides an alternate view of the characteristic inclusions.

Given this atypical imaging feature, NIID was suspected. Genetic testing for the NOTCH2NLC GGC repeat expansion returned negative. To establish a tissue diagnosis, a punch skin biopsy was obtained from the right calf. Electron microscopy revealed numerous round, non-membranous intranuclear inclusions within dermal fibroblasts, confirming NIID (Figures 2A, B).

The patient received supportive care and showed partial improvement in weakness by discharge. Because the final diagnosis was NIID rather than recurrent cerebral infarction, we discontinued long-term aspirin. This decision was based on three considerations: (1) NIID-related stroke-like episodes are now understood as “amyloid spells” secondary to uN2CpolyG cerebral amyloid angiopathy rather than atherothrombotic events; (2) no clinical guidelines recommend antiplatelet therapy for NIID; and (3) the patient had no conventional vascular risk factors and a mild-to-moderate isolated M2 stenosis, whereas her recurrent episodes with full recovery were characteristic of NIID. After shared decision-making, aspirin was stopped, and she remained event-free during 6 months of follow-up.

## 3 Discussion

This case provides several critical insights for vascular neurology practice, particularly in the evaluation of stroke mimics and complex cerebrovascular presentations.

### 3.1 The “ribbon sign”: a decisive neuroimaging clue in stroke mimics

The linear corticomedullary junction hyperintensity on DWI–termed the “ribbon sign”–is a hallmark of NIID that distinguishes it from common cerebrovascular or inflammatory leukoencephalopathies (1, 2). However, even in the presence of this sign, coexistent intracranial atherosclerosis (as seen in our patient) could mislead clinicians into attributing recurrent episodes to atherothrombotic events. This is because stroke-like episodes are a common manifestation of adult-onset NIID. As reported by Tian et al., stroke-like and encephalitis-like episodes, impaired consciousness, tremors, bradykinesia, limb weakness, bulbar palsy, and miosis are frequently observed in more than one-fifth of patients with NIID (3). Therefore, additional clinical features are critical for differentiation. In our patient, several clinical clues favored NIID over recurrent ischemic stroke. First, the temporal profile of the current episode was progressive worsening over 10 days (from mild weakness to hemiparesis), whereas acute ischemic stroke typically reaches its maximum deficit within seconds to minutes. Second, she lacked any conventional vascular risk factors (hypertension, diabetes, or hyperlipidemia). Third, the ipsilateral M2 stenosis was isolated and moderate (50% by MRA); however, MRA is not the gold standard for grading intracranial stenosis. MRA tends to overestimate stenosis severity compared to digital subtraction angiography (DSA), particularly in tortuous vessels or turbulent flow. Moreover, MRA cannot reliably characterize vessel wall pathology. Unfortunately, DSA was not performed in this patient, which is a limitation of our study. DSA would have provided a more accurate assessment of the true degree of stenosis, distal flow dynamics, and collateral status, and could have better clarified whether the M2 lesion was truly hemodynamically significant.

Thus, even when incidental atheromatosis is present, a clinical course characterized by subacute progressive onset, absence of vascular risk factors, and accompanying cognitive/autonomic features should prompt strong suspicion for NIID. Recognizing these clues allows clinicians to avoid misdiagnosis.

### 3.2 Navigating diagnostic limitations: beyond genetic testing

Although an expanded GGC repeat in NOTCH2NLC represents a major genetic cause of NIID, a substantial proportion of clinically and pathologically confirmed cases test negative with current methodologies (4, 5). Our case exemplifies this diagnostic limitation. In such situations, tissue biopsy becomes indispensable (6). Skin biopsy serves as a minimally invasive, high-yield diagnostic modality, reliably demonstrating intranuclear inclusions in fibroblasts and adipocytes (7). A pragmatic diagnostic pathway for stroke mimics with atypical features should therefore include skin biopsy when genetic testing is non-diagnostic yet clinical and imaging suspicion remains high.

### 3.3 Coincidence or connection? Revisiting NIID and cerebrovascular disease

The presence of significant MCA stenosis in our patient, who lacked conventional vascular risk factors, raises a compelling question regarding a potential link between NIID and accelerated intracranial atherosclerosis. NIID is a systemic proteinopathy known to affect vascular smooth muscle and endothelial cells. Chronic proteotoxic stress and low-grade inflammation associated with intranuclear inclusion formation may establish a pro-atherogenic microenvironment (8, 9). Analogous neurodegenerative-vascular interactions are increasingly recognized, as exemplified in Alzheimer’s disease (10). This hypothesis posits that NIID itself could act as a risk modifier for cerebrovascular disease, meriting further dedicated clinicopathological and imaging investigations.

## 4 Conclusion

Neuronal intranuclear inclusion disease represents an important though rare stroke mimic that may coincide with incidental intracranial stenosis. Vascular neurologists should recognize its pathognomonic “ribbon sign” on DWI. Achieving a definitive diagnosis through skin biopsy is crucial, as it fundamentally shifts long-term management from vascular secondary prevention to surveillance for neurodegenerative progression. This case underscores the necessity of an integrative diagnostic approach in patients with recurrent or atypical stroke-like presentations.

## Data Availability

The original contributions presented in this study are included in this article/supplementary material, further inquiries can be directed to the corresponding authors.
